# Melt-Spun P(3HB)/P(3HB-*co*-4HB) Monofilaments: Cyclic Loading Behavior and Fabrication into Textile Structures

**DOI:** 10.1021/acsomega.5c06548

**Published:** 2026-01-26

**Authors:** Sabrina Kopf, Sophie M. K. Hobrack, Dan Åkesson, Maria Persson, Mikael Skrifvars

**Affiliations:** † Swedish Centre of Resource Recovery, Faculty of Textiles, Engineering and Business, 1802 University of Borås, 501 90 Borås, Sweden; ‡ Department of Mechanical, Automotive and Aeronautical Engineering, Munich University of Applied Sciences, 80335 Munich, Germany; § The Swedish School of Textiles, Faculty of Textiles, Engineering and Business, University of Borås, 501 90 Borås, Sweden

## Abstract

Melt-spun polyhydroxyalkanoate monofilaments were successfully processed into woven and knitted textiles using industrial machinery, demonstrating their feasibility for textile applications. The filaments, composed of P­(3HB)/P­(3HB-*co*-4HB), exhibited an average tensile strength of ∼138 MPa and an elongation at break of ∼55%, with crystallinity of ∼30%. Cyclic loading of the filaments revealed pronounced hysteresis during the first cycle, which diminished in subsequent cycles. However, a relaxation time of 120 s was sufficient to reset the molecular conformational changes that occurred during the previous cycles. Furthermore, the incorporation of beta tricalcium phosphate (β-TCP) particles during melt spinning reduced tensile strength but improved thermal stability, enhancing processability. These findings highlight the potential of P­(3HB)/P­(3HB-*co*-4HB) monofilaments for sustainable textile applications requiring mechanical resilience and thermal robustness.

## Graphical abstract

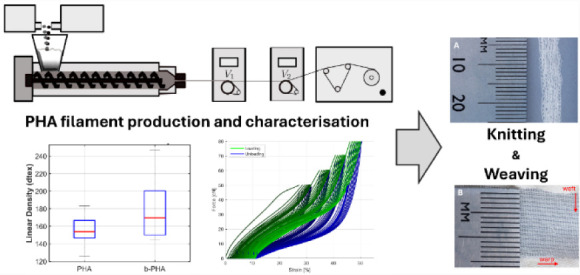

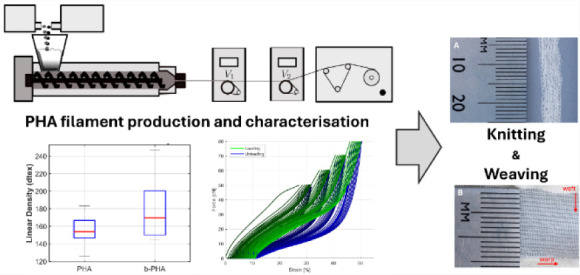

## Introduction

The textile industry is a significant contributor to global environmental burdens, accounting for more than 1.2 billion tonnes of CO_2_ emissions annually.^1^ Most synthetic fibers are made from fossil resources and are not biodegradable, which contributes to waste accumulation and microplastic pollution. Developing fibers that are both biobased and biodegradable could help address these issues and create more sustainable textile options.

Polyhydroxyalkanoates (PHAs) are a promising group of polymers because they are produced by microorganisms from renewable resources and can degrade in natural environments.^2^ PHA fibers are therefore of great interest not only for biomedical applications, such as sutures or scaffolds, but also for potential use in technical textiles, for example, agrotextiles.

However, PHAs are notoriously difficult to process. The most common polymer, poly­(3-hydroxybutyrate) P­(3HB), is brittle and has poor melt-processing properties. Its melting point is very close to its degradation temperature, which makes melt spinning challenging.^3,4^ These limitations have so far prevented P­(3HB) fibers from being widely used in textile applications. To overcome these challenges, new types of PHAs have been developed. One example is the copolymer poly­(3-hydroxybutyrate-*co*-4-hydroxybutyrate) P­(3HB-*co*-4HB), which shows improved elongation compared to P­(3HB).^5^ Microbial fermentation of this copolymer is under development.^6–9^ Omura et al. have reported melt spinning of P­(3HB-*co*-4HB) fibers,^10^ although these were thick laboratory-scale filaments primarily studied for marine biodegradability rather than for textile processing. In summary, this copolymer is promising, but to our knowledge, peer-reviewed demonstrations of successful conversion into woven or knitted textile have not been conducted. Thus, there is still a clear research gap in developing PHA filaments that combine reliable processing with realistic textile performance.

Beyond static tensile properties, fibers used in textile and biomedical applications are subjected to repeated deformation during use, which may lead to fatigue, loss of elasticity, or permanent deformation. In biomedical applications such as sutures, fibers are continuously exposed to cyclic stresses from body movement.^11,12^ Previous studies have shown that PHA-based fibers can exhibit elastic and partially reversible deformation under cyclic loading. Omura et al. demonstrated high elastic recovery of melt-spun P­(3HB-*co*-4HB) fibers after unloading,^13^ while Tsujimoto et al. investigated the mechanisms underlying the reversible deformation behavior of P­(3HB-*co*-4HB) fibers using in situ structural characterization.^14^ Perret et al. reported reversible orientation of highly oriented noncrystalline mesophases in melt-spun P­(3HB) fibers during cyclic tensile loading.^15^ While these studies provide important insight into the elastic response of PHA fibers, a complementary understanding of their cyclic mechanical behavior under application-relevant loading conditions remains important for assessing durability and functional performance, particularly with respect to the evolution of hysteresis, permanent deformation, and mechanical recovery under repeated loading.

In a recent study, we demonstrated that a blend of P­(3HB) and P­(3HB-*co*-4HB) could be melt-spun into filaments using a piston spinning machine.^16^ While this work confirmed the feasibility of producing continuous filaments, the long residence time in the piston limited fiber drawing and prevented further textile processing. Building on this work, the present study employs an extruder-based spinning setup to reduce degradation and, most importantly, demonstrates, for the first time, that the filaments can be processed into woven and knitted fabrics on pilot-scale textile equipment representative of industrial processes. In addition, to further explore their biomedical potential, bioactive fillers such as beta-tricalcium phosphate (β-TCP) were incorporated to enhance suitability for medical textile applications. This combination of mechanical testing and successful textile fabrication highlights a novel step toward making P­(3HB)/P­(3HB-*co*-4HB) fibers viable for both technical and biomedical applications.

## Material and Methods

### Filament Production

A polymer blend of 57 mol % P­(3HB) and 43 mol % P­(3HB-*co*-4HB) (30 mol % 4HB content) was used to spin the filament. The blend was supplied from Helian Polymers (Belfeld, The Netherlands) and processed using a Thermo Fisher Process 11 laboratory-scale twin-screw extruder (Karlsruhe, Germany). The temperature profile of the extruder, from the feeder to the circular die (diameter 1.0 mm), was set to 80, 165, 180, 190, 190, 190, 185, and 175 °C. The screw speed was set to 7 rpm, which resulted in a die pressure of 68 bar and a melt temperature of 171 °C. The formed extruded filament was drawn on two godets (Fourné Polymertechnik GmbH, Alfter, Germany) after exiting the die. Godet one was set to 42 °C and 9.0 m/min, and godet two to 28 °C and 70.0 m/min; both were positioned approximately 30 cm from the extruder outlet. The drawn filament was subsequently wound onto a bobbin using a precision take-up head (Barmag Spinnzwirn, Saurer GmbH & Co. KG, Chemnitz, Germany). The extrusion setup is shown in Figure 1.

**Figure 1:**
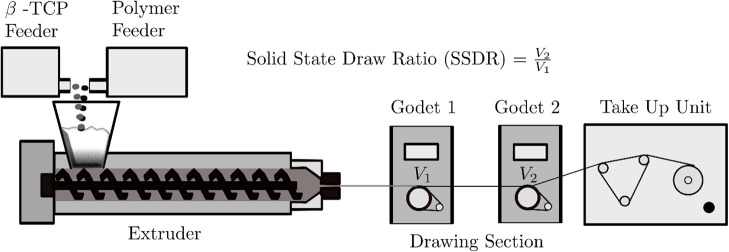
Schematic overview of the filament extrusion process and calculation of the melt and solid-state draw ratios.

Additionally, filaments with β-TCP particles (nanoXIM• TCP200 with a particle size of 5.0 ± 2.0 μm, used as received, Fluidinova, Maia, Portugal) were produced with the same setup. The temperature profile from the feeder to the circular die (diameter 1.0 mm) was adjusted to 80, 165, 180, 190, 190, 190, 185, and 165 °C. The screw speed was set to 20 rpm, resulting in a melt pressure of 37 bar and a melt temperature of 161 °C. For filament drawing, godet one was set to 42 °C at 10.0 m/min, whereas godet two was at 31 °C at a speed of 24.7 m/min. Furthermore, the godet pair was moved to approximately 85 cm from the extruder die. A gravimetric feeder was added to the extrusion setup for supplying the β-TCP. The feeder (Brabender GmbH & Co. KG, Duisburg, Germany) was calibrated to the bulk density of β-TCP (0.35 ± 0.10 g/cm^3^ according to the supplier) and discharged 0.035 kg of β-TCP in 1 h. The polymer and β-TCP were dried for 3 h at 80 °C in a vacuum oven prior to extrusion.

### Filament Analysis

#### Thermal Analysis

To verify the β-TCP filler content in the filaments, thermal gravimetric analysis (TGA; Q500, TA Instruments, Waters LLC, Wakefield, MA) was performed. Triplicate samples (*n* = 3) of filaments containing β-TCP (b-PHA) and those without (PHA) were equilibrated at 35 °C, then heated to 600 °C at a rate of 10.00 °C/min under a nitrogen flow rate of 60 mL/min.

Differential scanning calorimetry (DSC; Q2000, TA Instruments, Waters LLC, Wakefield, MA) was conducted in triplicate (*n* = 3) for both PHA and b-PHA filaments. Samples were first heated from room temperature to 200 °C and held isothermal for 5 min, followed by a cooling to −20 °C and a second heating to 200 °C. The heating and cooling rates for all cycles were 10.00 °C/min, under a constant nitrogen flow of 50 mL/min. To investigate the influence of the filament drawing, the degree of crystalline was calculated from both heating cycles using eq 1. Results from the first heating cycle were used to determine the degree of crystallinity after melt-and solid-state drawing, while the second heating cycle reflected the polymer’s inherent degree of crystallinity.

1 $$\chi_{P\left(\mathrm{3HB}\right)}=\frac{\Delta H_{m}\left(\mathrm{blend}\right)}{\Delta H_{c}\left(\infty \right)}$$

2 $$\chi_{\left(\mathrm{Polymer share}\right)}=\frac{{\Delta H}_{f}\left(\mathrm{blend}\right)}{\Delta H_{c}\left(\infty \right)}\times \frac{1}{w_{\mathrm{Polymer share}}}$$

where Δ*H*_c_(∞) = 146 J/g. In eq 1, Δ*H*_m_ represents the melting enthalpy, whereas Δ*H*_c_(∞) is the heat of fusion of the 100% crystalline polymer, in this case 146 J/g for P­(3HB). Equation 1 is used even though the filaments consist of a polymer blend because the P­(3HB-*co*-4HB) share is amorphous i.e., it does not crystallize. In eq 2, Δ*H*_f_(blend) and Δ*H*_c_(∞) represent the enthalpies of fusion of the blend and the 100% crystalline polymer, respectively, and ω_Polymer share_ represents the wt % of PHA in the blend.

#### Linear Density Distribution and Mechanical Analysis

The linear density, also known as titer or mass per unit length, is stated in tex (weight in g/1000 m filament) or dtex (weight in g/10,000 m filament) and was determined using a Favimat+ (Textechno, Mönchengladbach, Germany) via the nondestructive vibroscope method. In this method, the filament was subjected to sinusoidally alternating energy, which induced transverse vibrations. The fundamental resonant frequency of the filament vibration, in combination with the filament’s length and the applied pretension, was used to calculate the linear density. Results are reported in decitex (dtex), which corresponds to the mass of the filament in grams per 10,000 m of length. All tests were conducted with a 1200 cN load cell and a gauge length of 20.0 mm under standard climate conditions (21.9 °C, 73% relative humidity). The test speed was set to 20.0 mm/min with a pretension of 1.00 cN/tex.

For comparison, the linear density of as-spun PHA filaments, (i.e., the undrawn extruded filaments) was determined gravimetrically, as the filament fineness could not be measured using Favimat+. Prior to measurement, the as-spun filaments were conditioned for 2 days under the previously described climate conditions. The length of three samples was measured before the specimens were weighed, and the linear density was calculated in dtex using the measured mass and length.

The Favimat+ was also used for single-filament tensile testing. The test speed and pretension were set to 10.0 mm/min and 0.50 cN/tex, respectively. Linear density and tensile strength were evaluated for PHA (*n* = 20) and b-PHA (*n* = 10) filaments. First, the linear density of each filament was measured, followed by tensile testing of the same filament.

Additionally, as-spun filaments were tested using a universal testing machine (Universal H10KT, Tinus Olsen Ltd., Horsham, PA, USA) at a crosshead speed of 10.0 mm/min and a gauge length of 20.0 mm. Pneumatic grips with a clamp pressure of 1.5 bar were used to secure the samples during testing. A total number of 14 specimens were tested (*n* = 14).

To enable comparison of the tensile testing results obtained from both testing systems, it was necessary to approximate the filament diameter for the samples tested with the Favimat+. These calculations assume that the filaments exhibit ideal cylindrical geometry and were used to compute stress as a standard reference value. The cross-sectional area, *A* (cm^2^), was estimated using the following equation:

3 $$A=\frac{\mathrm{LD}}{\varrho \times 10^{6}}$$

where LD is the linear density in dtex, and ϱ is the polymer density in g/cm^3^. The density of the PHA blend was estimated to be approximately ϱ ≈ 1.31 g/cm^3^, based on volume measurements of the as-spun filaments.

##### Cyclic Loading

Three types of cyclic loading experiments were conducted. First, a force-controlled experiment was used to investigate the effect of testing velocity on the force-strain behavior of the filaments in order to identify potential time-dependent effects (*velocity variation experiments*). Second, cyclic loading was performed with progressively increasing maximum load levels to examine their influence on the displacement response (*maximum load variation experiments*). These data were also used to characterize the material’s elastic and plastic deformation behavior. Third, recovery time experiments were conducted to assess the effect of rest periods between loading cycles on the mechanical response (*recovery time experiments*). The objective was to identify time-reversible phenomena, such as hysteresis and plastic deformation, and to evaluate how filament behavior changes with increasing recovery time. This set of experiments enables a clear separation of rate effects, load dependent plasticity and time dependent recovery.

For most cyclic loading tests, ten loading–unloading cycles were conducted per parameter setting. Only the recovery time experiments were performed with five loading–unloading cycles to reduce the experimental time. Velocity variation and recovery time experiments were repeated five times whereas the maximum load variation experiments were repeated ten times. An overview of the experimental conditions is provided in Table 1.

**Table 1:** Parameter Settings for Each Type of Cyclic Loading Test

Cycle set (***n*** **= number of loading and unloading cycles in one set)**	**Force (cN)**	**Velocity** (mm/min)
Velocity variation experiments		
1, *n* = 10	50	10
	0	0
2, *n* = 10	50	10
	0	0
3, *n* = 10	50	100
	0	0
4, *n* = 10	50	150
	0	0
5, *n* = 10	50	200
	0	0
6, *n* = 10	50	10
	0	0
Maximum load variation experiments		
1, *n* = 10	50	50
2, *n* = 10	60	50
3, *n* = 10	70	50
4, *n* = 10	80	50
5, *n* = 10	50	50

**Cycle set (** *n* **= number of loading and unloading cycles in one set)**	**Force (cN)**	**Velocity** (mm/min)	**Rest period (s)**
Recovery time experiments			
1, *n* = 5	50	50	10
2, *n* = 5	50	50	20
3, *n* = 5	50	50	30
4, *n* = 5	50	50	60
5, *n* = 5	50	50	120
6, *n* = 5	50	50	240
7, *n* = 5	50	50	360

### Manufacturing Textile Fabrics

#### Knitting

The PHA monofilament was knitted into a single jersey tube using a Harry Lucas circular knitting machine (Maschinenfabrik Harry Lucas GmbH & Co. KG, Neumünster, Germany) equipped with 15 needles. The filaments were not treated with spin finish prior to knitting.

#### Weaving

A 1.5 cm wide and 15 cm long plain-woven PHA fabric was produced at 14 picks/cm on an SD1701 VAMATEX Jacquard weaving loom (Saurer Diedrichs) equipped with a Grosse electronic Jacquard unit. Fifty PHA filaments were used in the warp direction (33.3 ends/cm), and the PHA weft was fed using an IRO ORION M weft feeder (IRO AB, Ulricehamn, Sweden). No spin finish was applied, and the filaments were not sized before textile processing.

### Microscopy

A Nikon Eclipse LV100ND optical microscope and a 2.5× objective lens (Nikon, Japan) were used to obtain micrographs of the knitted and woven structures, while a 6.3× objective lens was used for the filaments. The associated software, NIS Elements BR, was used to measure the spacings between filaments and the diameter of the filaments.

### Statistical Analysis

Statistical analyses were performed using Minitab Statistical Software (Version 21.1.1) or MATLAB (R2024b). Group differences were evaluated using a one-way analysis of variance (ANOVA) followed by Tukey’s HSD post hoc test. Unless otherwise stated, statistical significance was set at α = 0.05. Data are reported as mean ± standard deviation, and the number of replicates (*n*) is stated for each data set. Differences in linear density were assessed using an unpaired two-tailed *t*-test with Welch’s correction for unequal variances. Results from the cyclic loading tests were compared using a paired *t*-test, with statistical significance set at *p* < 0.001.

## Results and Discussion

### Filament Production

In filament production, spinnability, defined as the ability to stretch polymers into long, continuous filaments without breakage, is a crucial factor. The processing parameters reported in the study represent the optimized conditions for the given experimental setup. For the PHA filaments, the extrusion and drawing processes were stable and free of spin-line breakages. The filaments could potentially be drawn further; however, the maximum godet speed limited additional drawing. Despite the absence of breakages, some filaments exhibited signs of draw resonance, an unstable spinning condition characterized by periodic variations in filament diameter.^17^ Draw resonance is typically caused by fluctuations in melt pressure and is more pronounced in materials with high melt elasticity. For example, in melt spinning of polyamide and polyethylene terephthalate blends, high melt elasticity was reported to contribute significantly to draw resonance.^18^ Given that the PHA blend in this study also exhibited high melt elasticity, it is likely that this behavior contributed to the observed instability. The high melt elasticity causes the polymer to elongate under drawing instead of flowing smoothly, resulting in melt accumulation at the die exit and occasional increases in filament thickness.

For the b-PHA filaments, the applied processing settings resulted in a stable spin-line (spinning for >30 min without spin-line breakage). However, any further increase in godet speed led to spin-line instabilities and, ultimately, filament breakage. The filaments primarily failed by fracture, also referred to as cohesive failure. Cohesive failure involves an instantaneous reduction of the filament’s cross section to zero and represents the upper limit of take-up velocity and spin draw ratio in an industrial setting. Beyond this point, successful spinning is no longer possible.^19,20^

The optimized processing parameters resulted in a solid-state draw ratio of 7.8 for the PHA and 2.5 for the b-PHA filaments. Filaments produced under these conditions are shown in Figure 2, where the addition of β-TCP not only increased the average filament thickness but also introduced greater variability in the fiber diameter. The PHA filaments had a fiber diameter of 128 ± 3 μm and the b-PHA filaments of 130 ± 6 μm which was the smallest diameter that could be produced under stable spinning conditions. This reduction in draw ratio for b-PHA is expected, as melt elasticity typically decreases with increased filler content.^21^ In the context of melt spinning, a spin draw ratio exceeding 20 is considered a critical threshold for isothermal spinning of Newtonian fluids, above which draw resonance is commonly observed.^20^

**Figure 2:**
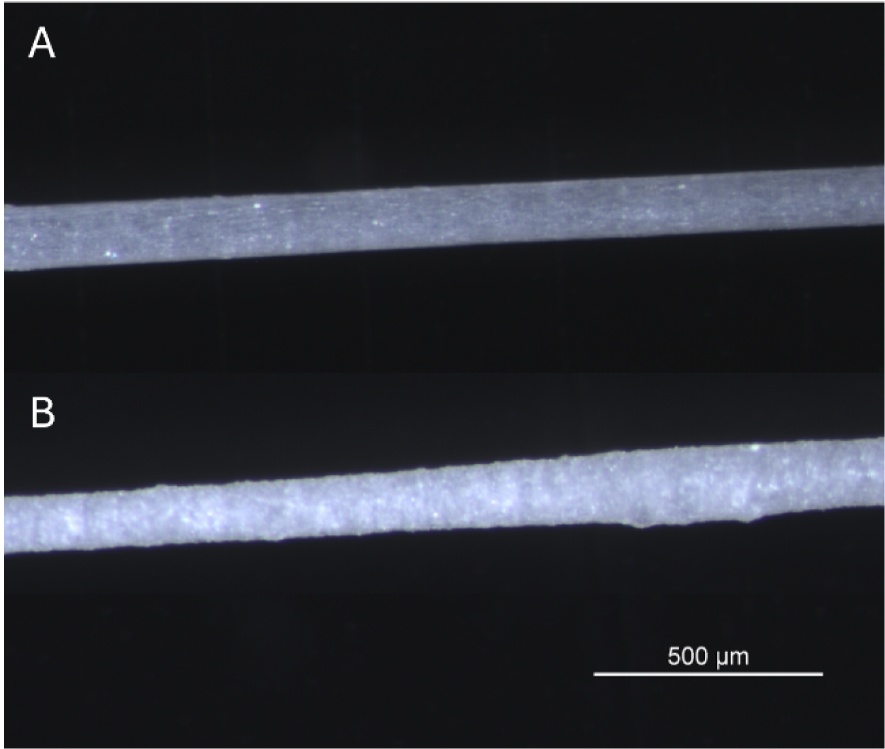
Optical microscopy image of the PHA (A) and b-PHA (B) filament.

### B-PHA Filler Content

The thermal properties and β-TCP filler content of the filaments were determined by TGA. A temperature range from 20 to 600 °C was selected, as β-TCP is thermostable within this range.^22^ Figure 3 shows the average thermograms of the b-PHA and PHA filaments. Both thermograms display a single degradation step and one sharp peak at the derivative weight curve, indicating rapid thermal degradation of the samples. The PHA filaments (Figure 3A) exhibit a residual weight of approximately 1.4%, while the b-PHA filaments (Figure 3B) show a residue of approximately 4.8%, corresponding to a β-TCP content of 3.4%. The 1.4% residue in the PHA filaments is attributed to a nucleation agent, which has a higher decomposition temperature and remains stable up to 600 °C.

**Figure 3:**
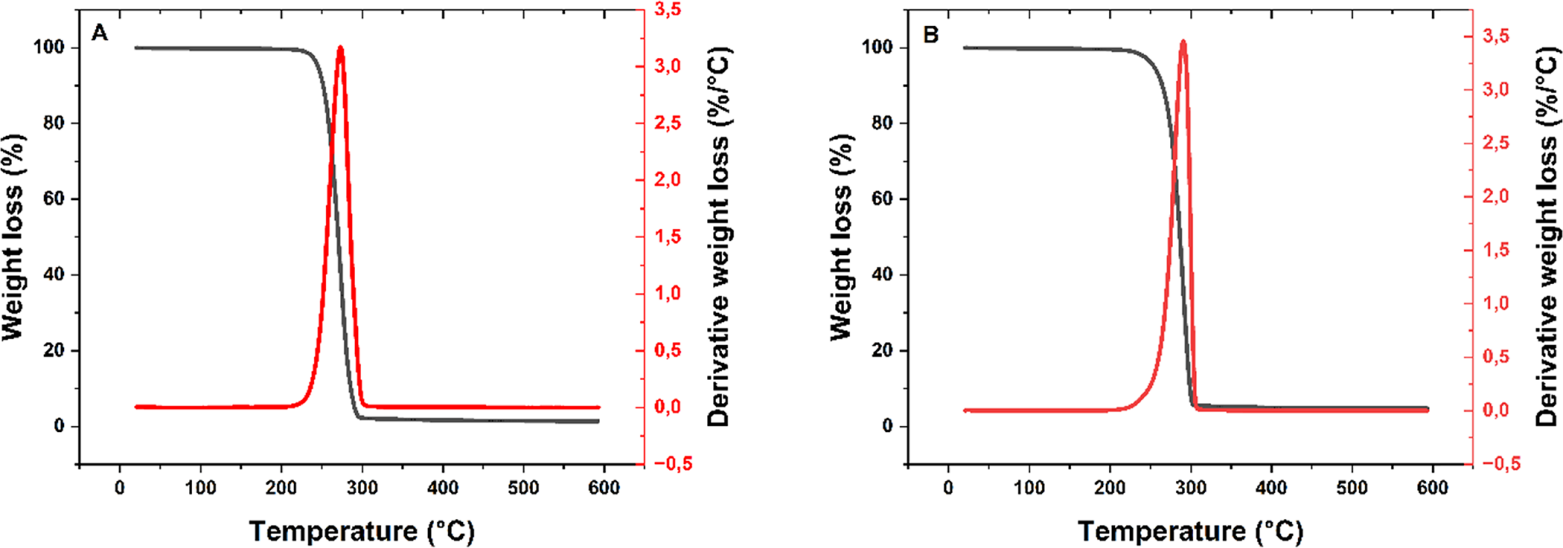
TGA curves for PHA (A) and b-PHA (B) filaments, averaged from three measurements (*n* = 3). The black line represents weight loss, and the red line represents the derivative weight loss. Both materials show a single degradation step with a sharp derivative peak, indicating rapid thermal degradation.

In addition to the filler content, the b-PHA samples also show a slightly higher thermal stability compared to the PHA samples, as indicated by the temperatures at 5% (*T*_5_) and 10% (*T*_10_) weight loss (Table 2). This increased stability is further reflected by a difference of approximately 15 °C in the temperature of maximum derivative weight loss, which corresponds to the maximum decomposition rate of the polymer.

**Table 2:** Overview of the Temperature of Five and Ten Percent Weight Loss as well as of the Maximum Derivative Weight and the Weight Residue at 600 °C for the PHA and B-PHA Samples (Mean ± Standard Deviation, *N* = 3)*T*

Sample	*T*_5_ (°C)	*T*_10_ (°C)	Weight residue at 600 °C (%)	Max deriv. weight loss (%/°C)
b-PHA	257 ± 1	267 ± 1	4.8 ± 0.05	293 ± 1
PHA	249 ± 5	256 ± 5	1.4 ± 0.02	277 ± 5

A one-way analysis of variance (ANOVA) at a 95% significance level was conducted to compare thermal stability parameters. The analysis revealed no significant difference between the b-PHA and PHA samples at *T*_5_ and *T*_10_, however, a significant difference was observed at the temperature of maximum derivative weight loss. This suggests that the presence of β-TCP influences the thermal stability of the polymer by shifting the main weight decomposition step to higher temperatures. In this case, the addition of β-TCP appears to enhance the thermal stability of the PHA, which is beneficial for melt processing.

### Melting and Crystallization Behavior

DSC was used to determine the influence of the filament drawing on the degree of crystallinity. The DSC thermographs of the PHA filament revealed a single melting and crystallization peak at 170 ± 0.04 °C and 113 ± 0.02 °C during the first heating and cooling cycle, respectively (Figure 4A). In the second heating cycle, two melting peaks were observed at 164 ± 0.09 °C and 174 ± 0.11 °C (Figure 4B). The b-PHA filaments exhibited similar thermal behavior, with one single melting peak at 170 ± 0.06 °C in the first heating cycle, a crystallization peak at 113 ± 0.07 °C, and two melting peaks at 163 ± 0.04 °C and 173 ± 0.04 °C in the second heating cycle. These filaments were composed of a polymer blend containing both an amorphous component P­(3HB-*co*-4HB) and a semicrystalline polymer P­(3HB). The melting peak at 170 °C was attributed to the P­(3HB) phase, consistent with values reported in the literature.^23^

**Figure 4:**
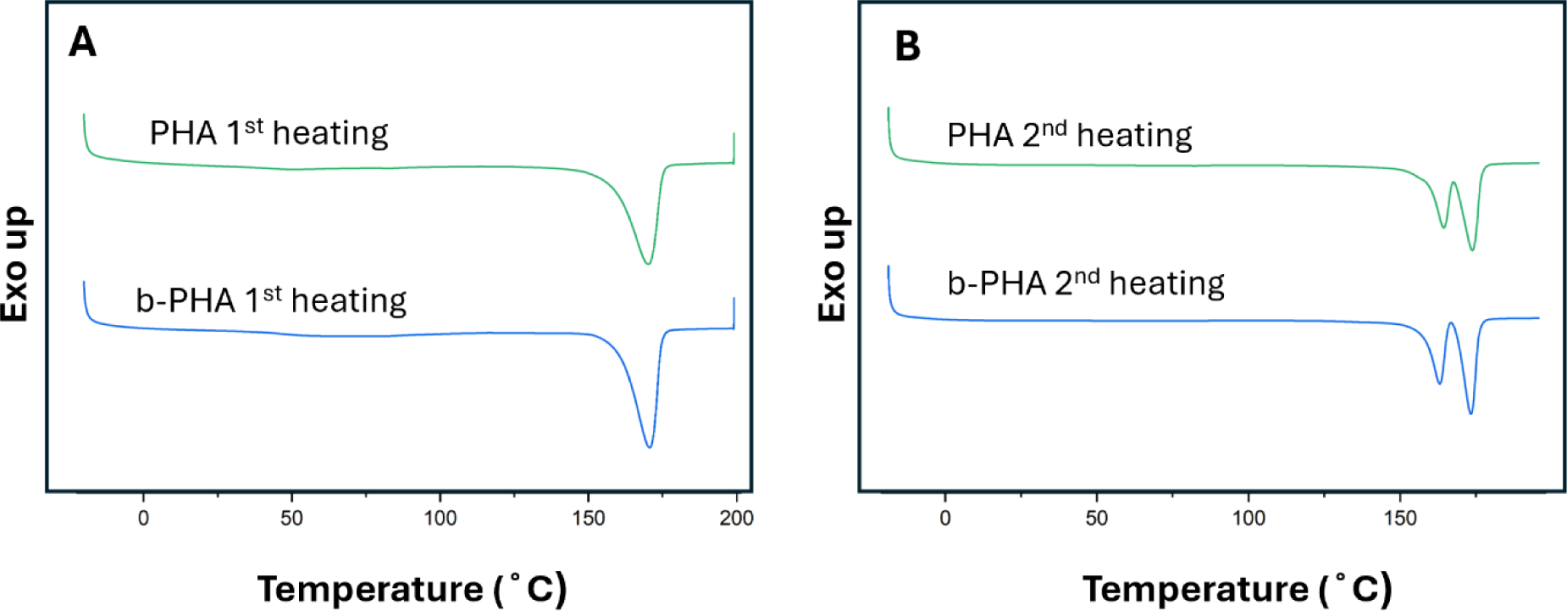
DSC thermograms of the first (A) and second (B) heating cycle of the PHA (green) and b-PHA (blue) filaments, based on the average of three measurements.

In general, P­(3HB) can form two crystalline structures: α and β. Helical α-chain configurations typically form upon melt cooling, whereas the zigzag β-form is generated under tensile stress, which induces recrystallization of the amorphous regions located between the α-crystal lamellae.^24,25^ Since the filaments in this study were subjected to drawing, formation of β-crystal could be expected. However, the β-crystal melting range is reported to be between 90 and 130 °C^26^ and no endothermic transitions were observed in this range in the DSC thermograms. This suggests that β-crystals were unlikely to have formed during filament processing and that the melting peak observed in the first heating cycle corresponds exclusively to α-crystalline structures.

The occurrence of multiple melting peaks for P­(3HB), as seen in the second heating cycle, is a well-documented phenomenon and can result from several reasons. These include (i) simultaneous melting–recrystallization and remelting, (ii) differences in crystal perfection, or (iii) polymorphism, where multiple crystal modifications coexist.^27^ In addition to thermal history, processing conditions such as temperature, pressure, and flow shear rate, as well as drawing and cooling conditions in filament spinning, can significantly influence melting behavior and crystalline structure.^27^

In the present study, a double melting peak appeared in the second heating cycle, during which the polymer was exposed to a controlled, load-free environment. This observation allows polymorphism to be ruled out as the cause. Previous research has shown that when P­(3HB) crystallizes isothermally at temperatures below 120 °C, the polymer shows two distinct melting peaks.^28^ While our samples were not crystallized under isothermal conditions, the crystallization peak occurred at 113 °C, which supports this explanation. The observed behavior is likely due to melting, recrystallization, and subsequent remelting. P­(3HB) can initially form crystals with lower thermal stability that melt at approximately 168 °C, then reorganize and melt again at higher temperatures around 172 °C.^29^

Drawing of filaments typically increases molecular orientation along the filament axis. This process improves tensile strength and recovery, influences the crystalline structure, and often increases the degree of crystallinity.^30^ However, in this study, the PHA filament showed a slightly lower degree of crystallinity in the first heating cycle as compared to the second (29.75% vs 32.08%). The first heating cycle reflects the filament’s thermal and mechanical history, including the drawing step. For semicrystalline polymers like P­(3HB), a similar or even a higher degree of crystallinity would typically be expected during the first heating cycle. The polymer blend used in this study appears to be at the threshold of semicrystalline classification. Polymers with a crystallinity of 30 to 35% or more are generally considered semicrystalline.^31^ Therefore, the observed values indicate that our filaments are predominantly amorphous. Furthermore, the presence of a melting peak indicates the existence of crystalline regions, which originate from the semicrystalline P­(3HB) component of the blend.

For the b-PHA filaments, there was no significant difference in the degree of crystallinity between the first and second heating cycles, both yielding approximately 32%. Interestingly, this is similar to the crystallinity of the PHA filaments, despite the b-PHA filaments being subjected to a lower draw ratio. This suggests that β-TCP functioned as a nucleating agent, enhancing the crystallinity of the b-PHA filaments, which aligns with previous findings.^18^

### Linear Density

Linear density is a crucial parameter in filament characterization, as it directly influences key mechanical properties such as stiffness and torsional rigidity.^32^ Accurate determination of linear density is essential for calculating stress–strain relationships and for normalizing tensile strength values, ensuring comparability across different filament samples. Additionally, it provides insight into filament uniformity and production consistency, allowing for quality control and process optimization efforts.^32^ Variations in linear density can lead to fluctuations in mechanical performance, impacting the reliability of the end-use applications such as textiles, composites, and biomedical applications.

Linear density measurements of the PHA and b-PHA fibers are presented in Figure 5. The inclusion of β-TCP resulted in a significant increase in the linear density of the fibers. While the pure PHA fibers exhibited a mean linear density of 154.7 ± 15.9 dtex, the b-PHA composite fibers showed a higher mean density of 178.4 ± 32.1 dtex (p = 0.049). Notably, the b-PHA group demonstrated considerably higher variability compared to the pristine PHA control.

**Figure 5:**
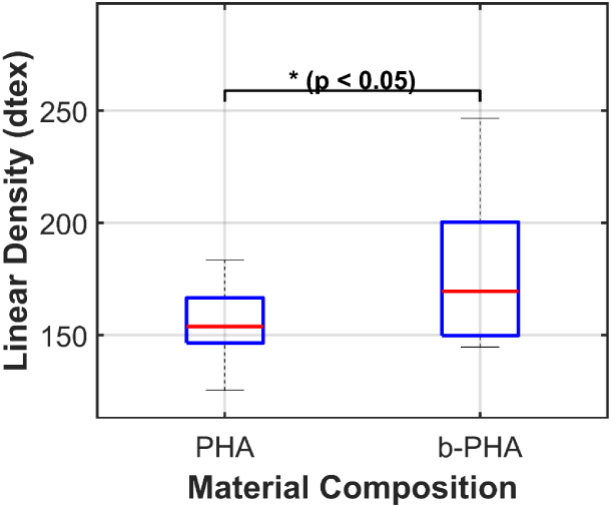
Boxplot of linear density measurements for PHA and b-PHA filaments (PHA: *n* = 20; b-PHA: *n* = 10). The graph highlights differences in variability and distribution between the two compositions. The boxplots display the median (red line), interquartile range (blue box), and whiskers (1.5 × IQR). The statistical analysis (unpaired *t*-test with Welch’s correction) confirms a significant difference between the groups (* *p* < 0.05).

Given that the PHA filaments had a solid-state draw ratio three times higher than that of the b-PHA filaments, a lower linear density was expected. Variations in linear density were reflected in the coefficient of variation (CV), which was calculated by dividing the standard deviation by the mean linear density and multiplying the results by 100 to express it as a percentage.^33^ The CV for PHA filaments was 5.3%, while that for b-PHA was 17.1%. For context, CV values for common man-made filaments range from 13.3% for Nylon (polyamide filament) to 21.4% for Terylene (polyethylene terephthalate filament).^34^ Based on these values, the linear density variability of both filament types is comparable to that of other synthetic fibers.

The b-PHA filaments showed a CV more than three times higher than that of the PHA filaments. In melt-spun fibers, such fluctuations typically originate from both rheological and mechanical factors. Rheological factors include phenomena such as draw resonance, die swelling, and melt fracture, while mechanical factors involve extruder output pressure, metering pump fluctuations, filament solidification conditions, and short-term disruption in the spinning line.^35,36^ In this case the primary contributor to the increased CV in the b-PHA filaments was the presence of β-TCP particles, which altered the rheological properties of the polymer. This resulted in a drop of the extruder’s output pressure and changed conditions of filament solidification. Particle agglomeration can also lead to fluctuations in the linear density and thus increase the standard deviation for the b-PHA filaments.

Additionally, the broader linear density distribution in the b-PHA filaments could be due to higher shear rates during extrusion. In thermoplastic materials, increased shear typically leads to a reduction in viscosity.^21^ The resulting viscosity drop caused a decline in melt pressure and required the first godet to be positioned further from the die to allow more time for solidification. In this setup, the distance between the die and the first godet was approximately 85 cm, increasing the filament’s exposure to potential short-term disruption such as vibrations, which could further affect spin-line stability and filament uniformity.

### Mechanical Analysis

#### Tensile Tests

Tensile tests were performed to evaluate the mechanical properties of the filaments. The force–displacement curves for both filament types are presented in Figure 6.

**Figure 6:**
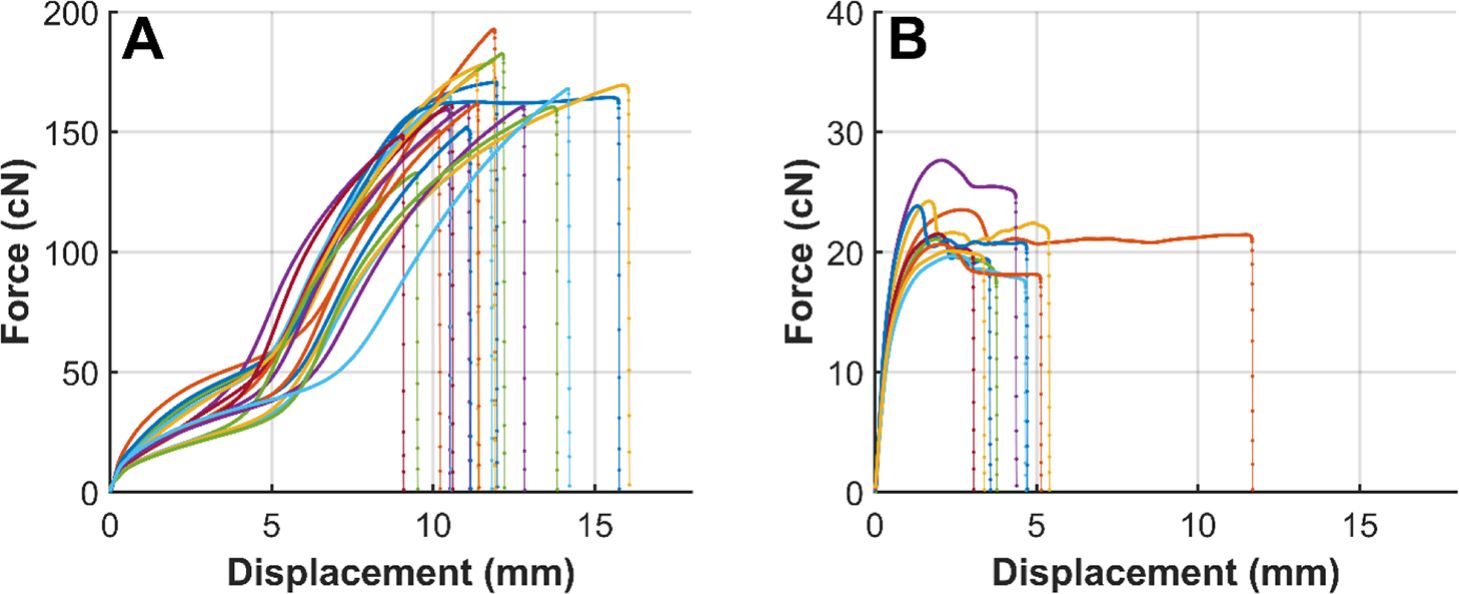
Force–displacement curves for PHA (A) and b-PHA (B) filaments (PHA: *n* = 20; b-PHA: *n* = 10).

PHA filaments displayed higher maximum loads (162.8 ± 13.7 cN), indicating greater tensile strength and the ability to undergo larger displacement before failure. As expected, b-PHA filaments demonstrated lower maximum force values (22.3 ± 2.4 cN) and a more brittle failure response, with substantially reduced displacement. The force plateau before failure was shorter for b-PHA, further reflecting reduced ductility compared with pure PHA filaments.

To account for differences in filament thickness, force–displacement curves were normalized by dividing the measured force by the linear density, resulting in specific stress values expressed in cN/tex (Figure 7). This normalization enables a direct comparison of mechanical performance across different filament samples, independent of their cross-sectional area. As anticipated, the normalized curves confirm that β-TCP containing filaments exhibited lower specific tensile strength (0.13 ± 0.02 cN/tex) compared with the PHA filaments (1.06 ± 0.1 cN/tex), indicating that the addition of β-TCP reduces the material’s load-bearing capacity. The reduction in tensile strength is attributed to interfacial debonding between the polymer matrix and the β-TCP particles.^37^ Without sufficient cohesion of particles and polymer, the polymer bears the majority of the load, while the particles act as defect sites, thereby decreasing tensile strength.^38^ Furthermore, the b-PHA filaments could not be drawn to the same extent as the PHA filaments, which limited the orientation of polymer chains in the b-PHA filaments and therefore contributed to their lower tensile strength.

**Figure 7:**
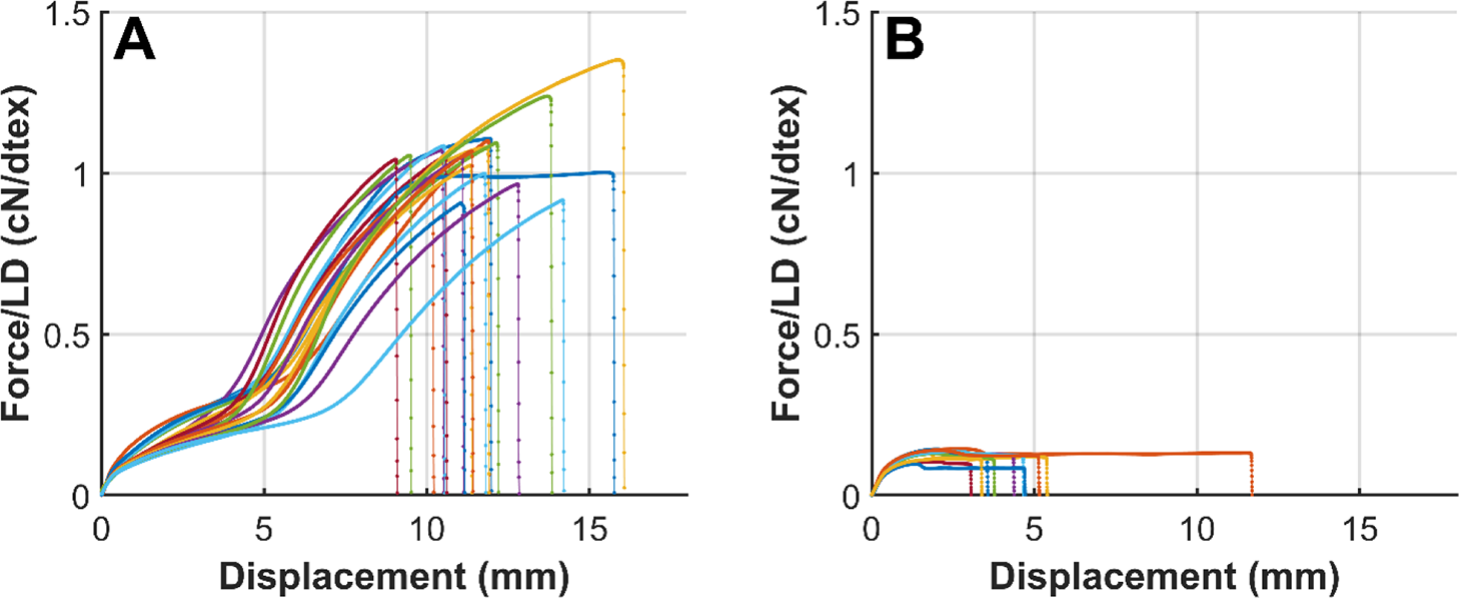
Normalized force–displacement curves for PHA filaments (A) and b-PHA (B), (PHA: *n* = 20; b-PHA: *n* = 10).

The ductility of b-PHA filaments is reduced because the β-TCP particles introduce stress concentration points that act as initiation sites for cracks and defects, leading to premature failure under tensile stress.^39^ Additionally, β-TCP can restrict the polymer chain mobility, which further reduces ductility.^40^ Different measures can be taken to mitigate the negative effects of the β-TCP particles on the tensile strength of the filaments. For instance, the particle size could be reduced, as this usually enhances the tensile strength of polymer composites.^41^ Alternatively, a surface modification of the β-TCP particles can be conducted to enhance the interfacial phase interaction of the polymer matrix and the β-TCP particles.^42^

A summary of key mechanical properties for each filament type is presented in Figure 8. The data indicate clear differences in mechanical behavior between the filament types. Drawn PHA filaments exhibited significantly higher maximum force (16.3 ± 1.4 N) compared to the b-PHA composite (0.22 ± 0.02 N, *p* < 0.001), confirming the detrimental effect of the filler on the tensile strength.

**Figure 8:**
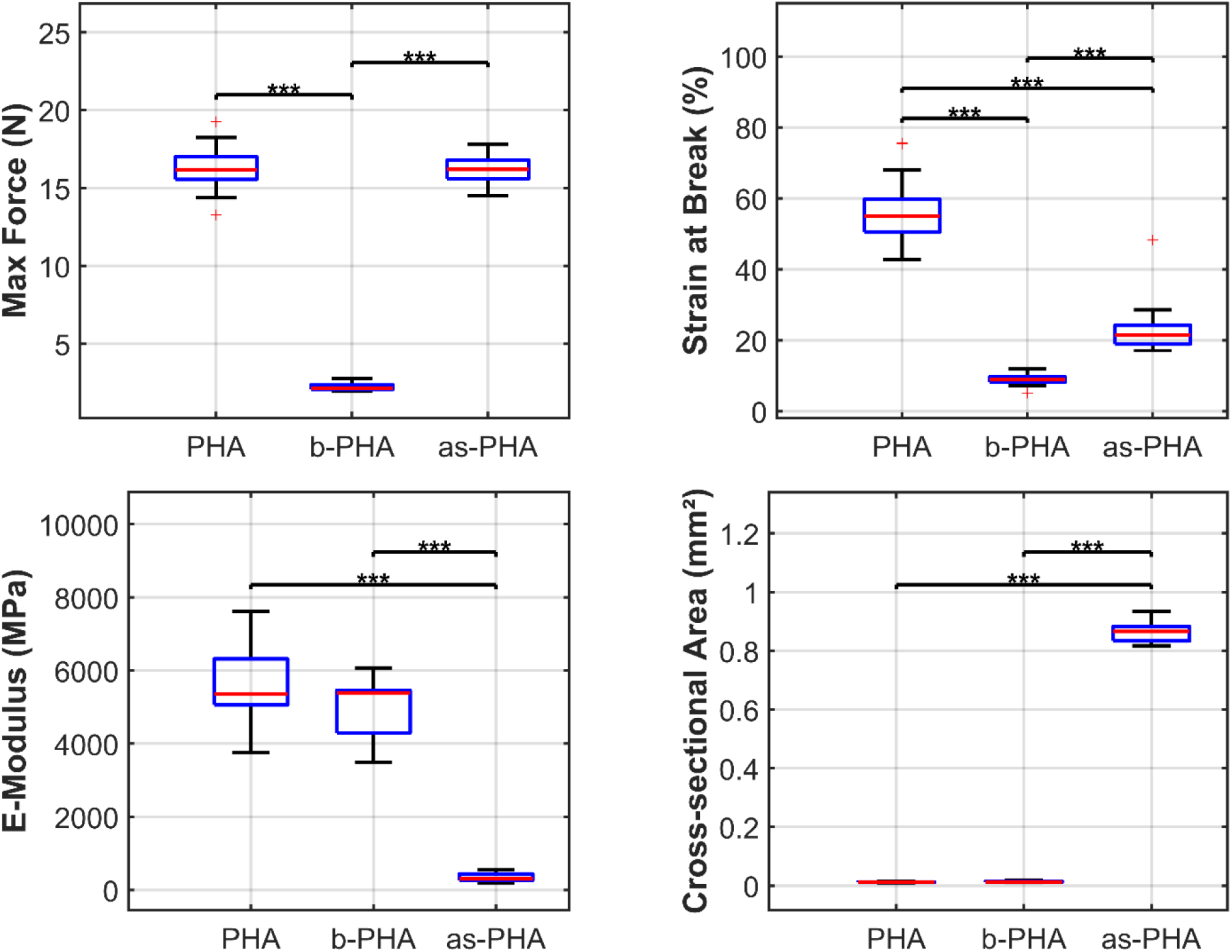
Comparison of mechanical properties of PHA, b-PHA and as-PHA filaments, (PHA: *n* = 20; b-PHA: *n* = 10, as-PHA: *n* = 14). (A) Maximum force at break, (B) Strain at break, (C) Young’s modulus, and (D) Cross-sectional area. The boxplots display the median (red line), interquartile range (blue box), and whiskers (1.5 × IQR). Statistical analysis was performed using one-way ANOVA followed by multiple comparison tests. Significant differences are indicated by brackets (*** *p* < 0.001).

PHA and as-PHA filaments withstood similar maximum forces, with no statistically significant difference observed between the two groups (*p* > 0.05). Considering the smaller apparent cross-sectional area of the PHA filaments, the effects of drawing and polymer chain alignment become evident. The as-spun filaments (as-PHA) had a significantly larger cross-sectional area (≈0.86 mm^2^) compared to the drawn PHA (≈0.012 mm^2^, *p* < 0.001). Consequently, when normalizing for dimensions, the drawn PHA filaments showed a significantly higher Young’s modulus (≈5400 MPa) compared to the as-spun material (≈320 MPa, *p* < 0.001), demonstrating the effectiveness of the drawing process in aligning the polymer chains and increasing stiffness. Statistical analysis of the elastic modulus did not reveal a significant difference between PHA and b-PHA filaments (*p* > 0.05); however, both were significantly different from as-PHA filaments (*p* < 0.05).

Although the filaments are predominantly amorphous based on their degree of crystallinity, the increased tensile strength of the drawn PHA filaments demonstrates that drawing induces molecular orientation. The effect of β-TCP on the tensile properties could not be fully evaluated due to processing limitations, which prevented the same drawing procedure for the PHA and b-PHA filaments. Nevertheless, clear differences in ductility were observed. PHA filaments without β-TCP demonstrated much higher ductility, with strain at break exceeding 50%, whereas β-TCP-containing filaments exhibited brittle behavior, with break strains around 10%. The total energy absorption before failure, i.e., work of fracture, was drastically reduced for β-TCP-reinforced filaments, suggesting a trade-off between strength and ductility.

These findings highlight the impact of β-TCP incorporation and filament drawing on mechanical performance, resulting in reduced tensile strength and ductility while maintaining comparable stiffness. The reduced energy absorption of β-TCP filaments suggests limitations for applications requiring high toughness.

The observed reductions in tensile strength, ductility, and work of fracture in the b-PHA filaments, containing relatively large particles (5 μm), align with previous studies on the effect of β-TCP particle size and concentration on the mechanical properties of thermoplastic polymers.^38^

#### Cyclic Loading

##### Velocity Variation

Force-controlled cyclic tests with a nonzero mean load were performed in sets of ten cycles per speed. The force was alternated between 0 and ≈50 cN while the actuator speed was stepped only between sets in the sequence 10 → 10 → 50 → 100 → 150 → 200 → 10 mm/min; the closing set at 10 mm/min serves as an internal check for reversibility and drift. Within each set the speed was held constant. Across this velocity window, the shape and area of the hysteresis loops remain practically unchanged and set medians of the hysteretic-energy equivalent vary only slightly; thus, the mechanical response is effectively rate-independent. The first set at a given speed shows a transiently broader spread that narrows with repetitions, consistent with short-lived viscoelastic conditioning rather than a rate effect. In the force-time traces, small apparent increases of the peak force at the highest speeds are caused by controller limitations/overshoot of the testing machine, not by material stiffening. A gradual right-shift with accumulated cycling indicates viscoelastic ratcheting, which is examined separately in the recovery-time study.

A representative curve of the velocity variation experiment is shown in Figure 9. The curves exhibit nonlinear behavior and are characterized by a pronounced hysteresis between loading and unloading. This hysteresis reflects energy dissipation during cyclic loading, which is typical for viscoelastic materials such as polymers. Ratcheting is confirmed by the shift of the hysteresis curves to a higher strain with increasing numbers of cyclic tests (Figure 9). This phenomenon arises from inelastic deformation caused by dislocation movements within the material, leading to the accumulation of permanent strain with each cycle.^43,44^ Considering the predominantly amorphous structure of the polymer, chain orientation and elongation under tensile load likely contribute to the observed permanent deformation.

**Figure 9:**
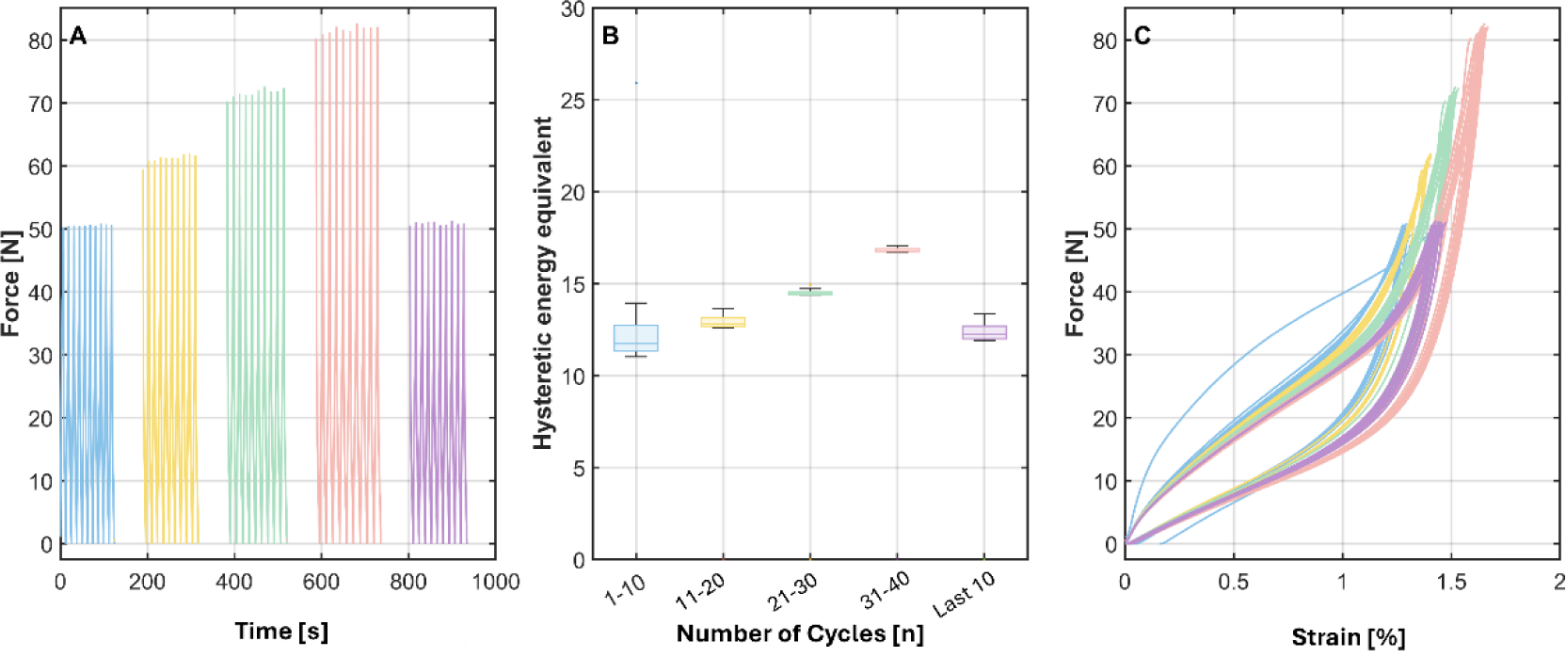
Representative curve of velocity-variation test under force control. Force–time history (A); boxplots of the hysteretic-energy equivalent per set (B); force–strain loops loading/unloading plotted separately (C). Colors denote speed sets in order (10, 10, 50, 100, 150, 200, 10 mm/min^1^) and are consistent across panels; within a set, lighter traces indicate individual cycles. Apparent peak-force rises at the highest speeds reflect controller overshoot, not a material effect.

The shift of the hysteresis curve with repeated loading has also been reported for P­(3HB-*co*-4HB) and P­(3HB) filaments, respectively.^13–15^ Consistent with the findings of Tsujimoto et al.,^14^ a residual strain was detected after the first cycle, which decreases over subsequent cycles. This behavior is attributed to the orientation and disentanglement of amorphous chain segments, which become more aligned and relaxed with continued cycles. Thus, the following cycles return to their original strain path without further residual deformation. The shape and area under the hysteresis curves remain constant across cycles, indicating that the test velocity does not influence the filament’s mechanical response. However, it appears that relaxation effects occur in the short break between each set of ten cycles, as the first cycle of every new set appears distinct. This behavior is unexpected given that the break between each set is less than 1 min. Despite this initial deviation, subsequent cycles within each series stabilize and exhibit comparable mechanical behavior. This consistent response across cycles suggests that the material adapts to cyclic loading, with minimal variations apart from the relaxation observed in the initial cycle of each new series.

##### Maximum Load Variation

Force-controlled cyclic tests were performed in sets of ten cycles per load level in the sequence 50 → 60 → 70 → 80 → 50 cN. Within each set, the loading rate was kept constant; only the maximum force changed between sets. The force-strain loops show pronounced hysteresis at all loads and a gradual right-shift with accumulated cycling, evidence of viscoelastic ratcheting. The hysteretic-energy equivalent is largest in the first cycle of a set and decays over subsequent cycles (short-lived viscoelastic conditioning), while set medians increase with maximum force. When the protocol returns to 50 cN, the loops and dissipation resemble those of the initial 50 cN set because the peak load is identical indicating that fast viscoelastic transients have largely relaxed during the intervening cycling. However, the loops remain slightly shifted to higher strain, and the energy does not fully match, consistent with incomplete recovery and slower viscoelastic/viscoplastic processes (e.g., chain orientation/disentanglement, microstructural softening). Overall, cyclic response scales with maximum load via strain amplitude and dissipation level, whereas loop shape within a set remains similar apart from the first-cycle transient.

For the cyclic loading with varying maximum loads, the same general material behavior was observed as in the velocity experiments (Figure 10). As seen in the previous strain-time curves from the velocity experiments, the strain accumulated progressively due to plastic deformation and shifted to higher levels over time. As expected, the strain amplitude increased with increasing applied force. However, in contrast to the velocity experiments, the maximum load variation experiment—where both the first and last set of cycles were conducted at a force of 50 cN—showed that the final set of cycles exhibited a higher strain than the initial one. This was likely due to the accumulation of plastic deformation under higher loads, which may have caused internal damage, such as microvoid formation or microcracks, leading to material softening.^45^

**Figure 10:**
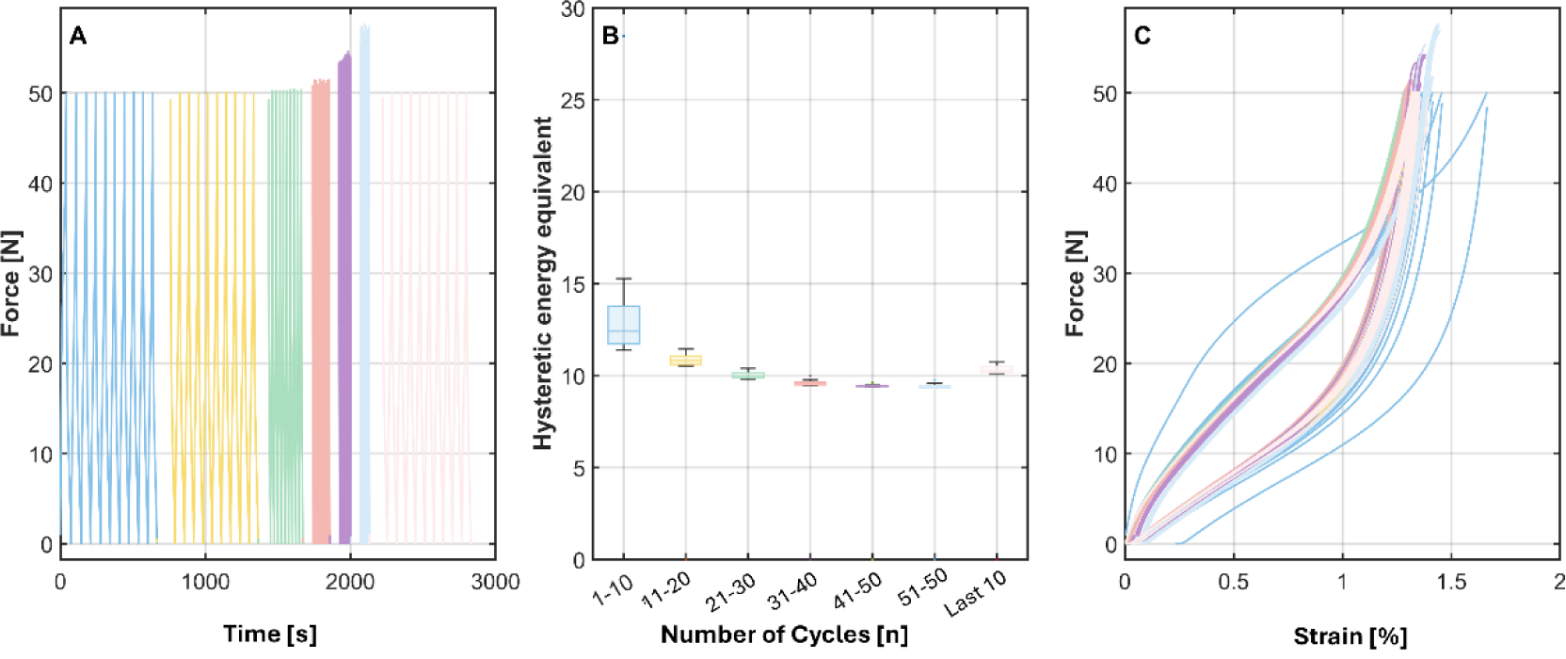
Representative graph of maximum-load variation under force control: ten cycles per set at 50, 60, 70, 80, and again 50 cN (A). Boxplots of the hysteretic-energy equivalent show larger dissipation at higher loads and a first-cycle transient (B). Force–strain loops of the final 50 cN set resemble the initial set (same load), reflecting partial relaxation of fast viscoelastic effects, yet remain slightly right-shifted, indicating slower processes and incomplete recovery (C).

This trend was also visible in the hysteresis loops, which shifted toward higher strain values with successive sets of cyclic loading. Notably, the first loading cycle of each set consistently deviated from the subsequent cycles. To quantify this effect, the hysteresis area for each loop and load level (i.e., 50 cN, 60 cN, 70 cN, 80 cN, and 50 cN) was calculated and is presented in Figure 11. The area under the hysteresis curve represents the energy dissipated through internal friction mechanisms in the polymer, such as viscoplastic deformation and molecular rearrangements.^46^

**Figure 11:**
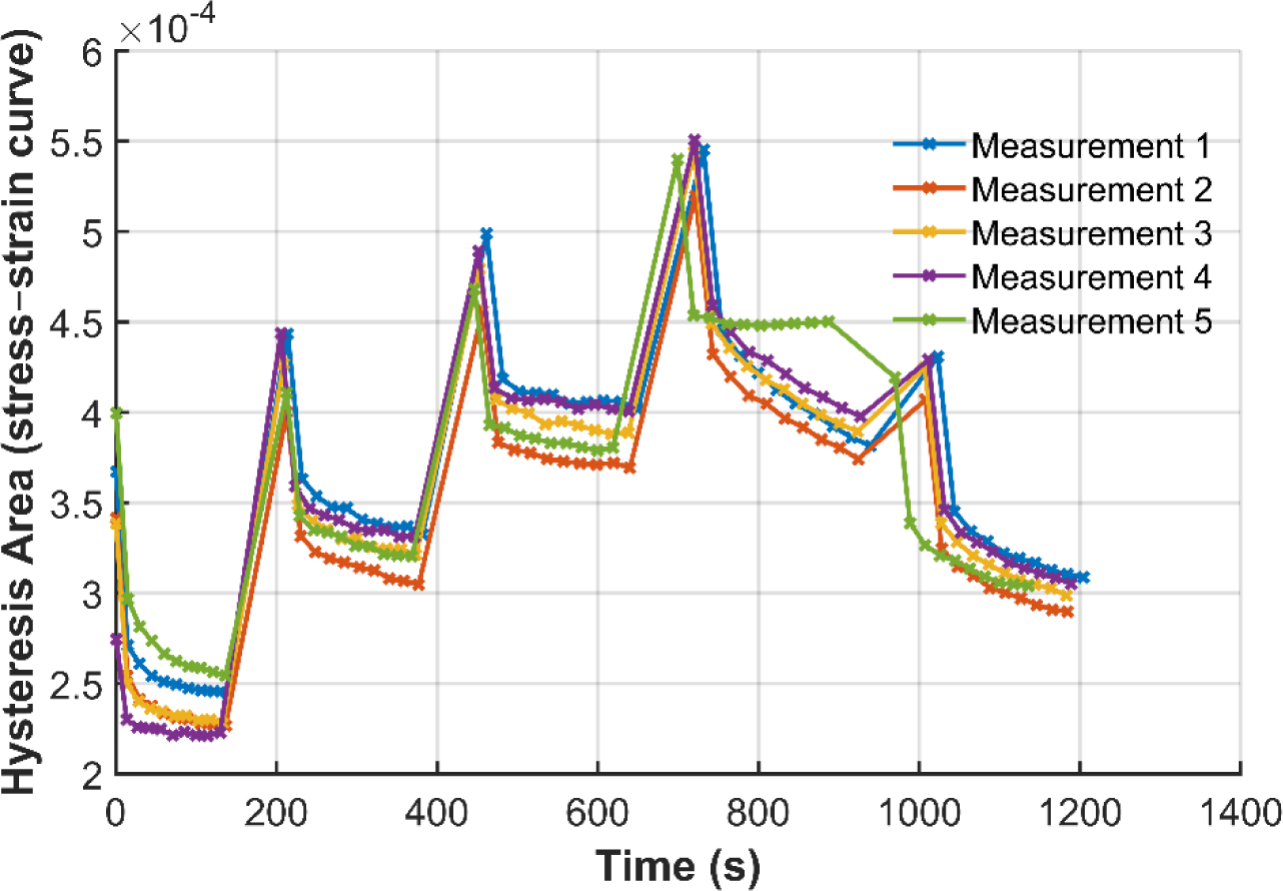
Representative plot of the hysteresis area over time of the maximum load variation experiments.

Hysteresis was most pronounced during the first cycle and gradually decreased in the subsequent loading cycles until the maximum load was increased for the next set of cycles (Figure 11). Although hysteresis increased with increasing maximum force, the overall phenomenon of a more distinct hysteresis in the first cycle followed by a decrease in the subsequent cycles remained consistent. Not all deformation was reversible, as the filament did not return to the initial force level. However, the graph suggests that viscoelastic recovery occurred during the 60 s break between sets of cycles. The maximum load variation experiments demonstrate that irreversible strain accumulation is governed by peak load, not by velocity. Higher residual strain observed after returning to the initial load level provides evidence of load-induced plastic deformation and microstructural changes such as chain orientation and disentanglement.

##### Recovery Time Variation

In the velocity variation experiments we observed a residual strain which was also noted by Tsujimoto et al.^43^ While Tsujimoto et al.,^43^ focused on investigating the elastic recovery rate and it is structural mechanisms, they did not examine the recovery time more closely. To further investigate the relaxation behavior of the filaments, cyclic loading tests with varying recovery times were conducted. Between sets of cycles, the filaments were allowed to rest for 10, 20, 30, 60, 120, 240, and 360 s, respectively. The hysteresis areas from these experiments are shown in Figure 12A. The largest hysteresis area was observed in the first cycle of the initial set, followed by a decrease in subsequent cycles within that set. After a short 10 s break between sets, partial relaxation occurred, as evidenced by the increase in hysteresis area in the first cycle of the second set compared to the last cycle of the first set. The hysteresis area then progressively decreased in the following cycles of the second set. This pattern continued in subsequent sets: the first cycle of each new set showed a slightly increased hysteresis area with longer relaxation times, followed by a gradual decrease in the remaining cycles of the set.

**Figure 12:**
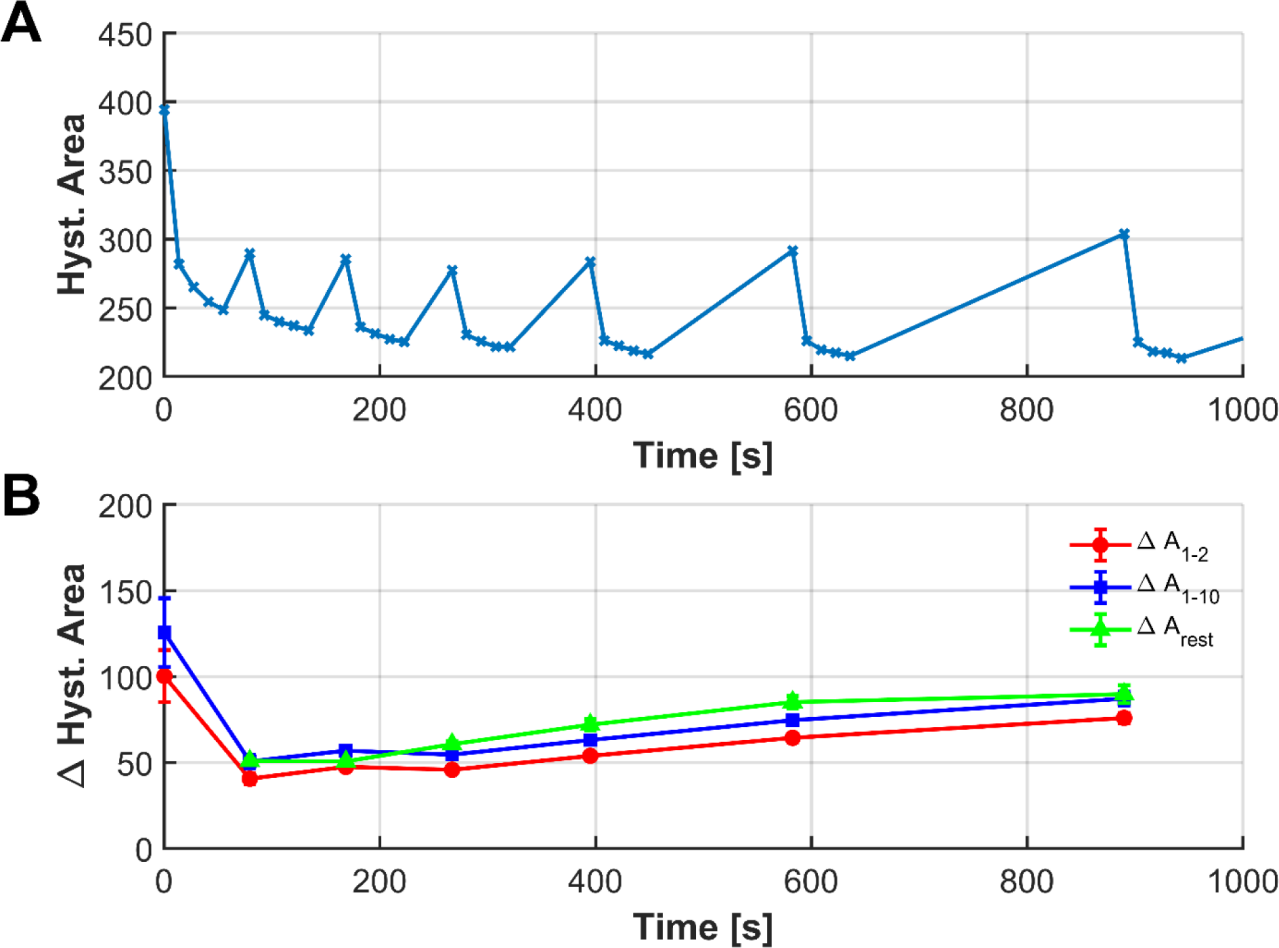
Evolution of hysteresis area during cyclic loading with varying recovery times (A). Hysteresis area for tests with rest periods of 10–360 s between cycle sets (B). Changes in hysteresis area over time: red (Δ*A*_1–2_) and blue (Δ*A*_1–10_) denote differences within sets, while green (ΔA_rest) shows changes between sets Data represents mean ± standard deviation (*n* = 5). The convergence of the green and blue curves after approximately 120 s indicates near-complete structural recovery, and the increase in recovery capacity (ΔA_rest) over the experiment was confirmed to be statistically significant (*p* < 0.001, paired *t*-test).

Large hysteresis areas indicate high energy dissipation and strong viscoelastic behavior, whereas small hysteresis areas are typical for more elastic materials with minimal internal energy loss. Thus, a decrease in hysteresis area suggests reduced energy dissipation, primarily due to reduced internal friction and viscoelastic losses.^46^ Considering that the filament is approximately 70% amorphous, the majority of the polymer chains are not highly ordered. Hence, the initial filament loading probably orients the amorphous regions located between crystalline domains,^47^ where high internal friction leads to large hysteresis areas in the early cycles. As the chains become increasingly aligned in the tensile direction, internal friction decreases, resulting in reduced energy dissipation in subsequent cycles.

Cyclic loading was performed at room temperature, which is above the glass transition temperature of the PHA blend. This allows the polymer chains in the amorphous phase to rearrange during the recovery periods. Short breaks of 10 s were sufficient to initiate this relaxation process, during which chains returned toward a random coil configuration. Consequently, the first cycle of each new set encountered increased resistance, leading to a temporary rise in hysteresis area, as observed in Figure 12.

The changes in the hysteresis area over time are shown in Figure 12B. The red curve represents the difference between the first and second cycles within each set of ten cycles, while the blue curve shows the difference between the first and last cycles within the set. Both curves follow the same trend, indicating that the largest difference occurs between the first and the second cycle, i.e., most of the energy is introduced during the first cycle. The green curve represents changes in the area during the resting periods, specifically the difference between the last cycle of one set and the first cycle of the following set. Over time, the green curve converges with the blue curve; at a hold time of 120 s, the differences in hysteresis area are nearly identical. This suggests that the molecular changes induced during the cycle set were largely reset during the resting period. To verify the significance of the observed recovery behavior, a statistical analysis was performed on the hysteresis data (Figure 12B). The plot displays the mean differences in hysteresis area with error bars representing the standard deviation across five independent measurements (*n* = 5). While the structural changes within a cycle set (red and blue lines) stabilize quickly, the recovery of energy capacity (green line, ΔA_rest) shows a distinct increase with longer rest periods. A paired *t*-test confirmed that the recovery after the final rest period was statistically significantly higher than after the first rest period (comparing the interval after the first cycle set vs the last cycle set), with the mean recovered energy increasing from 50.9 to 89.7 (*p* < 0.001). This validates the time-dependent relaxation capability of the polymer chains.

In summary, the recovery time experiments provide direct mechanistic insight by identifying a characteristic recovery time scale: most viscoelastic losses occur during the first cycle, and the filament largely recovers within approximately 2 min at room temperature.

### Manufacturing Textile Fabrics

The PHA filament was processed on industrial-scale machines. Both circular knitting and weaving with a rapier loom were possible and led to the successful fabrication of two textile structures (Figure 13).

**Figure 13:**
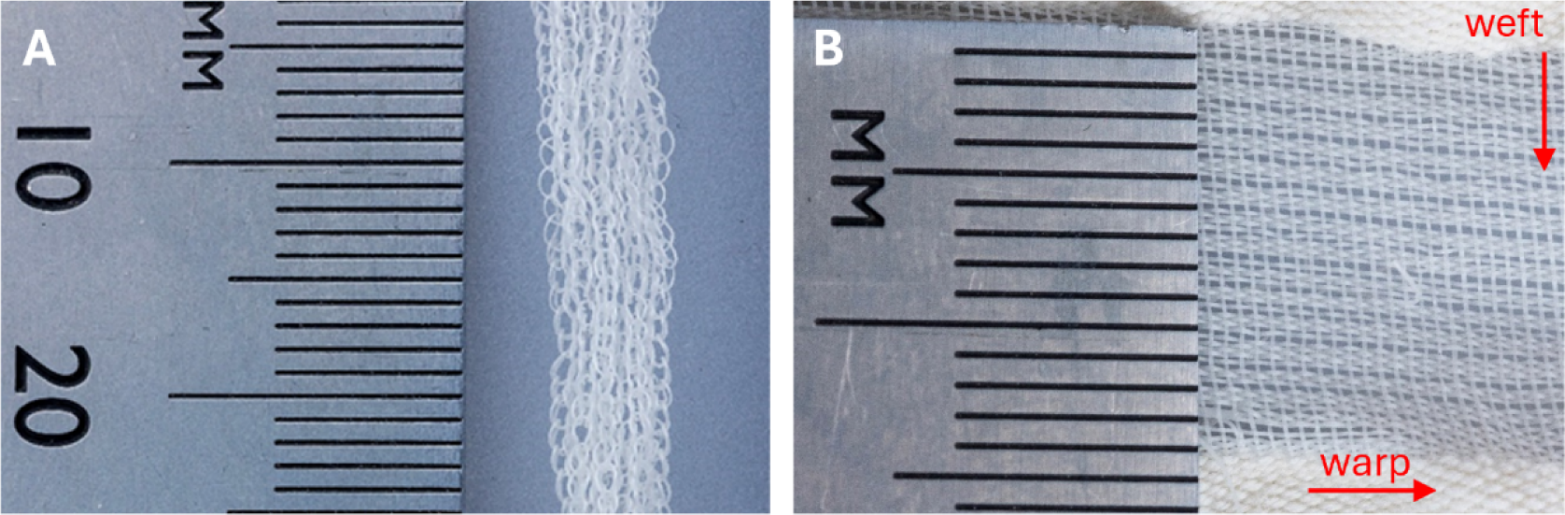
Photographs of the knitted (A) and woven (B) textile.

Producing different textile structures is of practical relevance because the filament experiences various types of stress during processing. Weaving is the process of interlacing two sets of yarns—warp (longitudinal direction) and weft (transverse)—to create a fabric. For knitting, needles are used to form interlocking loops of yarn to create a fabric. In both processing methods, the filaments are exposed to frictional forces when gliding along the parts of the machinery. In weaving, the filament is additionally exposed to friction between the warp and weft yarn. Tensile forces are applied in knitting during the loop formation and as the filament is pulled through the knitting machine. However, tensile loads in knitting are relatively low, making filament elongation and elasticity more critical than tensile strength.^48^ In weaving, the filament is exposed to constant or cyclic loads in the warp direction^49^ at the weaving machine to maintain the fabric structure. In knitting, the filament is especially exposed to shear and bending forces during the loop formation, especially in the filament’s cross-section when the filament is drawn through the needle’s hook, whereas shear forces are present in weaving during filament interactions with component parts like drop wires. Being able to produce fabrics from the PHA filaments showed that they can withstand the different forces they are exposed to during processing.

For imaging purposes, the knitted tube was stretched to the maximum to reduce the fabric’s bulkiness. In the stretched state, the length of the loops is ca. 724 μm, and the width at the widest part is around 250 μm. The spacings in the woven structure are smaller and range from ca. 53 to 205 μm. These are only examples of pore sizes and can be altered to suit the intended application, depending on the filament’s thickness and machine gauge.

## Conclusions

Melt spinning is a major industrial process for the manufacturing of man-made fibers, producing more than half of the synthetic fibers produced globally. Even though the experimental setup in this study is simplified in contrast to industrial-scale melt spinning that enables more precise control of the filament formation by melt pumps, quenching, and the application of spin finish, this setup can be used to get a first indication of which fiber properties can be obtained by the polymer blend.

In this study, a polymer blend of semicrystalline P­(3HB) and amorphous P­(3HB-*co*-4HB) was successfully melt-spun into monofilaments and processed into both knitted and woven textiles. The resulting filaments were predominantly amorphous, with a degree of crystallinity of approximately 30%, and exhibited elastic properties. Recurrent variations in filament thickness were observed, attributed to the high melt elasticity, which led to draw resonance during spinning. Cyclic loading tests revealed ratcheting behavior in the PHA filaments, i.e., inelastic deformation progressively accumulated in the material. Hysteresis was most pronounced in the first loading cycle and decreased in subsequent cycles within each set. This decreasing trend indicated reduced energy dissipation over time, reflecting the material’s elastic behavior. The high energy dissipation in the first cycle was likely due to the orientation of the amorphous phase; once aligned, the polymer dissipated less energy unless allowed to recover. Recovery time experiments showed that the filaments regained their initial mechanical response within 120 s, suggesting that the amorphous chains relaxed and returned to a coiled configuration. Furthermore, β-TCP particles were incorporated during melt spinning. Their inclusion led to a decrease in tensile strength and ductility, attributed to stress concentration effects, restricted polymer chain mobility, and insufficient particle–matrix interaction. Considering the intended application field in medical textiles for bone reconstruction, the filaments show some benefits. The average tenacity of the fibers (∼138 MPa) is close to the tensile strength of cortical bone (125 MPa). Furthermore, the Young’s modulus of the fibers is around 5 GPa, which falls within the range of bone (0.5 to 30 GPa, depending on the type of bone and the person’s age). In this regard, the relatively low crystallinity of the filaments is also advantageous. Regions with low crystallinity can act as reservoirs for active ingredients in controlled drug-release applications, making the fibers promising candidates for medical textile use. However, to verify the applicability of the fibers for the mentioned medical applications, further tests are required. Furthermore, it is interesting to investigate not only the tribological properties of the fabrics but also modeling the mechanical behavior of the filament and textile structure.
